# Molecular Shells and Range of Interactions in Ionic Liquids as a Function of Temperature

**DOI:** 10.1021/acs.jpclett.4c03576

**Published:** 2025-02-20

**Authors:** Zhiyuan Gao, Florin Teleanu, Kelsey Anne Marr, Alexej Jerschow

**Affiliations:** Department of Chemistry, New York University, New York, New York 10003, United States

## Abstract

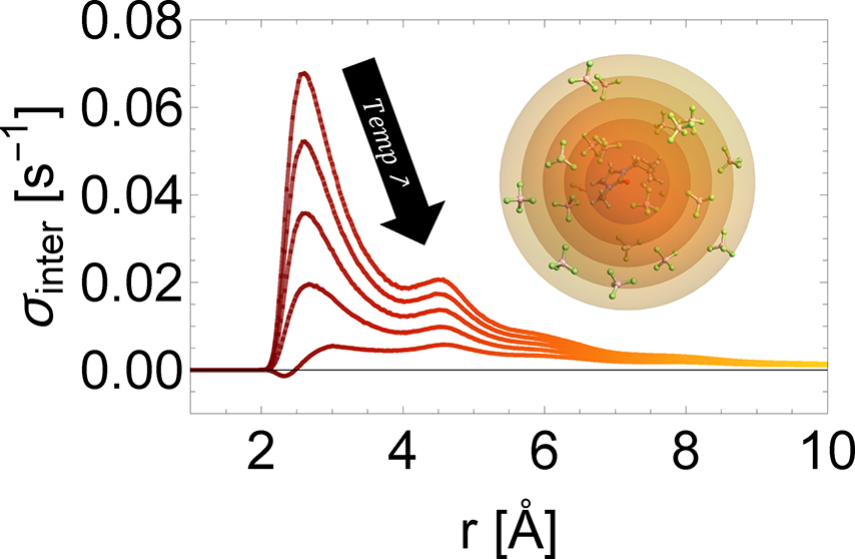

Room-temperature ionic liquids (RTILs) represent a versatile class of chemical systems composed entirely of oppositely charged species whose bulk properties can be fine-tuned by adjusting molecular structures and, consequently, intermolecular interactions. Understanding the intricate dynamics between ionic species can aid in the rational design of RTILs for specific applications in a range of fields, including catalysis and electrochemistry. Here, we investigate the temperature dependence of intermolecular interactions through magnetization transfer by means of ^1^H–^19^F heteronuclear Overhauser effect spectroscopy (HOESY) for two ionic liquids, namely, [BMIM][BF_4_] and [BMIM][PF_6_]. We find that the cross-relaxation rates vary significantly over a rather small temperature range, even changing sign. Molecular dynamics (MD) simulations on neat RTIL systems replicate this behavior well and further show that the dynamic properties rather than coordination changes of RTILs account for the observed temperature behavior. Furthermore, the investigation of different coordination shells highlights the change of interaction range with temperature even to the point where inner and outer coordination shells could be in distinct motional regimes with cross-relaxation rates of opposite sign. Since temperature changes lead primarily to dynamic changes rather than structural ones, these findings underscore the versatility and high thermal stability of ionic liquids.

Ionic liquids (ILs) make up a distinct class of solvents characterized due to their unique molecular structure and versatile properties. ILs are typically composed of bulky organic cations and either inorganic or organic anions.^1^ These substances are prized for their remarkable properties, which include high thermal stability,^2^ high ionic conductivity,^3^ negligible vapor pressure, and excellent solvation capabilities for a wide range of materials. Many investigated ILs are in the liquid phase at temperatures below 100 °C,^2^ even often at room temperature. These properties make them highly useful as electrolytes in batteries,^4,5^ solvents for both organic and inorganic compounds in chemical synthesis,^6^ and drug delivery matrices.^7^

Nuclear Magnetic Resonance (NMR) spectroscopy is a powerful analytical technique to investigate molecular structures, environments, and dynamics of ILs in the liquid phase at the atomic level.^8^ Various NMR techniques have been developed to study ILs including relaxometry,^9^ diffusometry,^10−12^ and polarization transfer via the Nuclear Overhauser Effect (NOE)^13−16^ from which ion proximity, viscosity, conductivity, and translational and rotational diffusion can be deduced. NMR observables such as chemical shifts, scalar coupling constants, or autorelaxation rates are less affected by temperature, making polarization transfer a promising tool to study the dynamics and especially intermolecular interactions in RTILs. The Overhauser effect can stem from both intra- and intermolecular dipolar interactions among spins, showing different scaling laws with respect to the distance between nuclei.^17^ In the case of intramolecular dipole–dipole coupling, the polarization transfer depends on the molecular rotational correlation time and the internuclear distance *r*, which for a rigid molecule makes the Overhauser effect proportional to *r*^–6^. The intramolecular NOE is usually considered effective up to a distance of 5 Å^18^ for ^1^H spin pairs (less for heteronuclear pairs). For intermolecular transfer,^19^ the distance between spins varies significantly during molecular collisions and only transiently becomes smaller than 5 Å. Nonetheless, the polarization pool comprises many more spins at larger distances (farther than 5 Å)^20^ for which dipole–dipole interactions decorrelate slowly, resulting in long correlation times and rendering the effective cross-relaxation rate proportional to *r*^–1^. This phenomenon makes the intermolecular NOE transfer not only adequate, but also highly informative for studying sub-ns dynamics in viscous systems such as ILs^14−16^ being a significantly more affordable alternative to neutron scattering techniques.^21,22^

Heteronuclear Overhauser Effect Spectroscopy (HOESY) involves transient NOE transfer among different types of nuclei, such as ^1^H–^13^C, ^1^H–^15^N, or, as in this work, ^1^H–^19^F. HOESY experiments have become a powerful tool in the study of molecular structures where different types of atoms are in close proximity. The efficiency of the Overhauser effect in heteronuclear systems is a function of several factors including the distance between the two nuclei, their relaxation properties, molecular tumbling, and the inherent differences in their magnetic properties such as the gyromagnetic ratio. Here, we use HOESY techniques to monitor the polarization transfer from the chemically and magnetically equivalent ^19^F spins located on the inorganic anions to the different ^1^H spins located on the organic cations. We chose two imidazolium-based ILs, namely, 1-Butyl-3-methylimidazolium tetrafluoroborate ([BMIM][BF_4_]) and the hexafluorophosphate analogue ([BMIM][PF_6_]). Based on the relative strength of the intermolecular Overhauser effect, one can map the spatial distribution of the anions around the cations, locating the sites with strong Coulombic attractions. For this purpose, we used the pulse sequence^23^ shown in Figure 1a and monitored the ^1^H signal evolution as a function of the mixing time τ_*mix*_. The strength of the NOE can be quantified by fitting the build-up curves and extracting the cross-relaxation rates σ. Deriving the Solomon equations^24^ for a system of two dipolar-coupled heteronuclear spin-half nuclei, we obtain the following expression^25^ for the build-up curve:
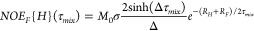
1where *NOE*_*F*_{*H*} stands for the signal intensity of a chosen ^1^H peak originating from the transient NOE induced by ^19^F nuclei after a mixing time τ_*mix*_. *M*_0_ is a normalization factor^25^ related to the Boltzmann equilibrium magnetization of ^1^H and ^19^F, σ is the cross-relaxation rate, , and *R*_*H*_ and *R*_*F*_ are the spin–lattice relaxation rates of proton and fluorine spins, which were measured by inversion recovery experiments (see Figure S1).

**Figure 1:**
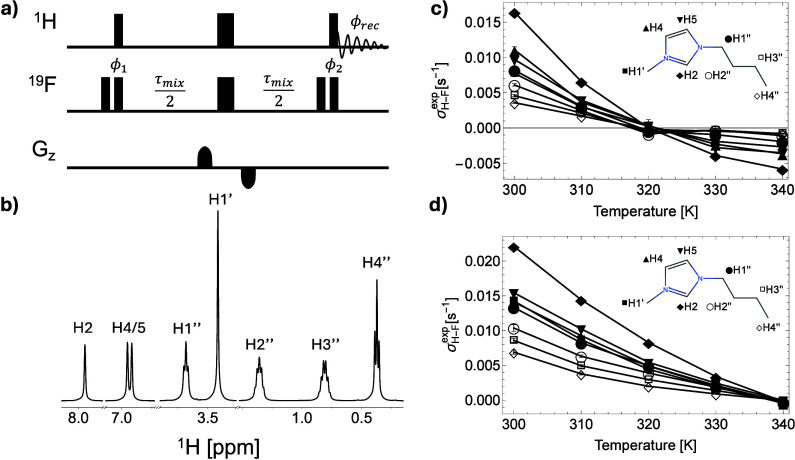
(a) The 1D HOESY pulse sequence for measuring ^1^H–^19^F intermolecular polarization transfer. The narrow and wide rectangles represent nonselective 90° and 180° pulses, respectively. The choice of phase cycling and homospoil bipolar gradients ensures that only NOE transfers are measured. The 4-step phase cycle is ϕ_1_ = [*x* – *x x* – *x*]; ϕ_2_ = [*x x* – *x* – *x*]; ϕ_*rec*_ = [*x* – *x x* – *x*]. (b) 1D ^1^H spectrum of [BMIM][BF_4_] with assigned resonances (see the atom labeling on the right). (c and d) Fitted ^1^H–^19^F intermolecular cross-relaxation rates for [BMIM][BF_4_] and [BMIM][PF_6_], respectively, at different temperatures. The protons bonded to the carbon atoms in the same plane as the imidazolium ring are shown with filled markers, while the ones in the mobile part of the *n*-butyl arm are shown with empty markers. Error bars were calculated using the covariance matrix of the parameter estimation from fitting build-up curves in Figure S2 using eq 1, where σ_*H*–*F*_^*exp*^ was the only fitting parameter.

*NOE Build-up Curves and Relative Order of σ.* The pulse sequence of Figure 1a is designed to invert the ^19^F spins and detect the transient NOE on the ^1^H spins (further details in the Methods section). Since each manifold of chemically equivalent protons in the organic cation showed a well-resolved peak (Figure 1b), we were able to measure polarization transfer from anions to all individual protons in the BMIM cation. Figures 1c and 1d show the fitted cross-relaxation rates for the labeled protons at different temperatures, the trends of which are discussed below.

The NOE build-up curves provide essential insights into the spatial relationships between the fluorine nuclei of the anions and the protons of the cations. At short mixing times, the NOE signal builds up linearly within the first second of the mixing time and decays exponentially thereafter. The experimental build-up curves were fitted very well with Equation 1 where the cross-relaxation rate was the only fitting parameter (see Figure S2). As a first observation, a strong correlation is noticed between the relative magnitudes of σ and the chemical shift values of the corresponding protons: nuclei that resonate at lower fields also have larger σ values. One could explain a part of this correlation by the fact that higher partial positive charges lead to stronger counterion attraction but would also lead to enhanced relative deshielding (peaks at higher ppm values). Of course, an additional factor would be ring current effects, which will be stronger for protons closer to the imidazolium ring (more deshielding on the ring and closer to the ring than further away).^26^

The intensity of the intermolecular NOE signal is strongly influenced by the distribution of distances between the interacting nuclei, as it is inversely proportional to the internuclear distance (*r*^–1^). Consequently, shorter intermolecular distances result in steeper NOE build-up curves and larger σ values. Following the trends in Figure 1, a proximity map of the fluorinated anions around the organic cation can be established. The results indicate that the BF_4_^–^ and PF_6_^–^ anions exhibit stronger attraction to the nitrogen atoms of the imidazolium ring, likely due to the delocalized positive charge within the aromatic system. This interaction positions the anions closer to the protons on the imidazolium ring while distancing them from the protons on the butyl side chain, as shown by the relative magnitudes of the cross-relaxation rate (Figure 1c and 1d).

*The Temperature Dependence of σ*. We first observe the interesting trend that the cross-relaxation rates all cross zero at almost the same temperature (320 K for BF_4_ and 340 K for PF_6_). This result indicates that intramolecular reorientation, such as side chain motion, does not significantly affect intermolecular interactions. Furthermore, it is somewhat intuitive that the rates for the PF_6^-^_ case would cross zero at larger temperatures than for BF_4^-^_ due to the larger size and slower diffusion.

Based on the relative magnitude of the cross-relaxation rates, the relative distances between (^1^H) and (^19^F) nuclei remain consistent across temperatures, indicating no significant structural changes in the spatial arrangement of cations and anions in the ionic liquids within the studied temperature range. However, all σ decrease and even switch signs as the temperature increases: the cross-relaxation rates simultaneously cross the null point at 320 and 340 K for [BMIM][BF_4_] and [BMIM][PF_6_] systems, respectively. As the Overhauser effect involves population transfer by both zero-quantum and double-quantum transitions, we examined how temperature might impact the probability of these two phenomena. For our case, the heteronuclear cross relaxation rate can be expressed as σ = 0.6*J*_*inter*_(ω_*H*_ + ω_*F*_) – 0.1*J*_*inter*_(ω_*H*_ – ω_*F*_) where ω_*H*_/2π = 400.228 MHz and ω_*F*_/2π = 376.729 MHz are the Larmor frequencies of proton and fluorine spins at 9.4 T, respectively, and *J*_*inter*_(ω) is the frequency-dependent spectral density characterizing the fluctuations generated by the relative tumbling of ions. The first term gives the probability of double quantum (flip–flip) transitions, while the latter characterizes the zero-quantum (flip–flop) transitions, their relative contributions dictating the sign and amplitude of σ. Specifically, the polarization transfer takes place from the sum of ^19^F longitudinal operators to individual ^1^H longitudinal operators solely due to the pairwise ^19^F–^1^H dipolar interactions. To check for potential effects of cross-correlation, we used the automated symbolic processing of Bloch–Redfield–Wangsness relaxation theory workflow^27^ to compute the analytical expression for the cross-relaxation rate in a system of one ^1^H and four ^19^F spins where all dipole–dipole (DD) and chemical shielding anisotropy (CSA) interactions were considered as well as their cross-correlations (see Supporting Information). The computed rate proves that the polarization transfer does not involve any cross-correlated mechanisms and stems from the sum of independent pairs of ^19^F–^1^H dipole–dipole contributions.

The expression of the intermolecular spectral density is given as^19,28^
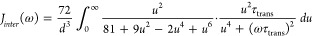
2where the translational correlation time is expressed as τ_*trans*_ = *d*^2^/*D*_*HF*_ with *d* being the distance of closest approach and *D*_*HF*_ = *D*_*H*_ + *D*_*F*_ is the sum of the self-diffusivity for the cation and anion, respectively. The origin of the temperature profile of σ can arise from changes in either structural or dynamical factors.^29^ As the distance of closest approach is not immediately accessible by NMR experiments, being an ensemble average, rather than a fixed distance between two atoms of the same molecule, we performed Molecular Dynamics (MD) simulations and computed the pairwise radial distribution function (RDF) between protons and fluorine atoms as well as the mean square displacement (MSD) of ions (Figures 2a,b and 3a,b). It is shown that the RDF changes only slightly with temperature, while the slopes of the MSD plots increase significantly. These results suggest that it is the self-diffusivity rather than interionic distances that lead to changes in the intermolecular spectral densities: faster translational diffusion leads to shorter correlation times τ_*trans*_ which modulates the relative contributions of the zero- and double-quantum transitions.

**Figure 2:**
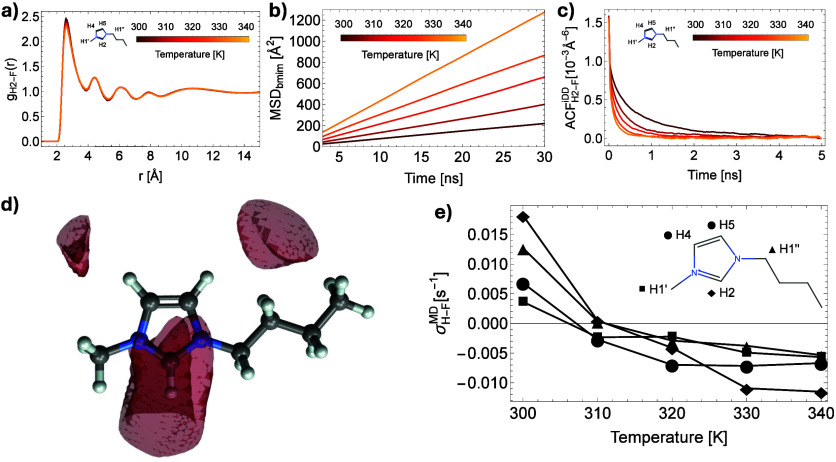
Molecular Dynamics simulations of neat [BMIM][BF_4_] systems with the thermostat set to temperatures between 300 and 340 K. (a) Radial Distribution Function (RDF) for the H2-F pair at different temperatures (proton labeling shown as inset). (b) Mean Square Displacement (MSD) for the BMIM cation at different temperatures. (c) Time-evolution of the autocorrelation function *G*(*t*) corresponding to the intermolecular dipolar coupling between H2 protons and fluorine atoms. (d) Spatial Distribution Function (SDF) isosurfaces of fluorine atoms around BMIM cations at 300 K. (e) Intermolecular ^1^H–^19^F cross-relaxation rates computed from MD trajectories at different temperatures for protons bonded to the carbon atoms lying in the same plane.

**Figure 3:**
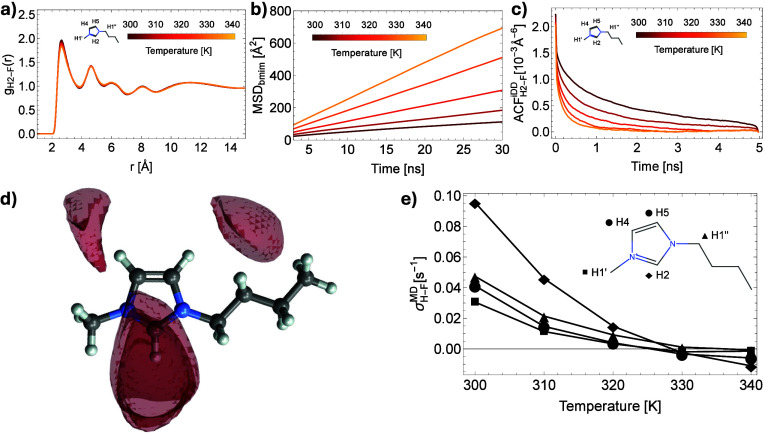
Molecular Dynamics simulations of neat [BMIM][PF_6_] systems with the thermostat set to temperatures between 300 and 340 K. (a) Radial Distribution Function (RDF) for the H2–F pair at different temperatures (proton labeling shown as inset). (b) Mean Square Displacement (MSD) for the BMIM cation at different temperatures. (c) Time-evolution of the autocorrelation function *G*(*t*) corresponding to the intermolecular dipolar coupling between H2 protons and fluorine atoms. (d) Spatial Distribution Function (SDF) isosurfaces of fluorine atoms around bmim cations at 300 K. (e) Intermolecular ^1^H–^19^F cross-relaxation rates computed from MD trajectories at different temperatures for protons bonded to the carbon atoms located in the same plane.

To support our interpretation, we calculated the intermolecular cross relaxation rates from MD trajectories and compared them to those observed experimentally. The spectral density function is proportional to the Fourier transform of the autocorrelation function characterizing the intermolecular dipolar coupling *G*(*t*) given as *J*_*inter*_(ω) = *K*_*HF*_∫_0_^*∞*^*cos*(*ωt*)*G*(*t*)*dt* where  and
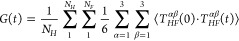
3where the dipole–dipole tensor components *T*_*HF*_^*αβ*^ are
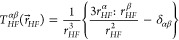
4with  being the time-dependent vector between ^1^H–^19^F spin pairs, *N*_*H*_ and *N*_*F*_ the number of proton and fluorine atoms considered, and α, β the axes labels (*x*, *y*, *z*). Thus, the autocorrelation function *G*(*t*) measures how fast the intermolecular ^1^H–^19^F dipolar interaction decorrelates during molecular tumbling. Starting from a chosen BMIM residue, we calculated the time-dependent ensemble averages ⟨*T*_*HF*_^*αβ*^(0)·*T*_*HF*_^*αβ*^(*t*)⟩ for all ^1^H–^19^F spin pairs within a 12 Å distance and then averaged over a number of 100 cation systems. Figures 2c and 3c show the averaged autocorrelation function *G*(*t*) describing the intermolecular dipolar interaction. Generally, as the thermostat in our simulations is set to higher temperatures, the loss of correlation is faster, signifying a shorter translational correlation time τ_*trans*_. Self-diffusivity for both ions also increases with temperature. To examine the relative contributions of different factors, we compared the computed self-diffusivity rates of both ions (extracted by fitting MSD plots) with experimental values obtained by either pulsed-field-gradient spin–echo^10^ or field cycling relaxometry^9^ and obtained a very good match (see Figure S3).

One significant observation is that the structural or chemical aspects of the anionic species do not appear to be major factors in the observed trends, as the null point of the cross-relaxation rates for both [BMIM][BF_4_] and [BMIM][PF_6_] corresponds to the temperature for which the sum of self-diffusivities is around 8 × 10^–11^ m^2^/s. This is in agreement with the similar position of the first peak in the RDF plots for the two ILs, around 2.58 Å, leading to a translational correlation time of τ_*trans*_ ≈ 800 ps for which the cross-relaxation rate is zero. This is in very good agreement with translational correlation times extracted from field-cycling relaxometry at similar temperatures.^9^

Furthermore, we analyze the relative intensities of the cross-relaxation rates among the BMIM’s ^1^H spin system obtained from our simulations (Figures 2e and 3e). The largest σ values correspond to protons on the imidazolium ring where most of the positive charge is delocalized (H2, H4, and H5 in Figure 2e and 3e), followed by the ones of the methylene and methyl groups (H1″ and H1′). As these protons are bonded to carbon atoms lying in the same plane as the imidazolium ring, they experience similar translational diffusion rates. Thus, the relative rate of cross-relaxation is dictated by the (averaged) spatial proximity of the fluorine nuclei, which can be easily inspected by visualizing the spatial distribution function (SDF) in Figures 2d and 3d. As expected, protons in closer contact with fluorine nuclei, due to their higher partial positive charge, will also show higher cross-relaxation rates.

Lastly, we investigate the shell-resolved contributions of surrounding spins to the observed cross-relaxation rate. This is achieved by computing first the spectral density profiles using eq 2 and the measured self-diffusion rate at variable temperatures and fixed intermolecular distances (see Figure 4a,b). Only the contributions fluctuating at the sum and difference of the ^1^H–^19^F Larmor frequencies at 9.4 T account for the relaxation phenomena observed in our experiments. The temperature and distance dependence of intermolecular cross-relaxation rate highlights that the close shell contributions are dominant and very sensitive to temperature changes, while the more distant shells have a smaller, but constant, contribution (Figure 4c). The temperature profiles of σ_*inter*_ at fixed distances exhibit a decreasing trend, followed by a sign change and eventual asymptotic behavior approaching zero (Figure 4d). These findings can be attributed to changes in diffusion rates and, consequently, to changes in the translational correlation times as well as σ being inversely proportional to distance, which has a probabilistic distribution rather than being fixed.

**Figure 4:**
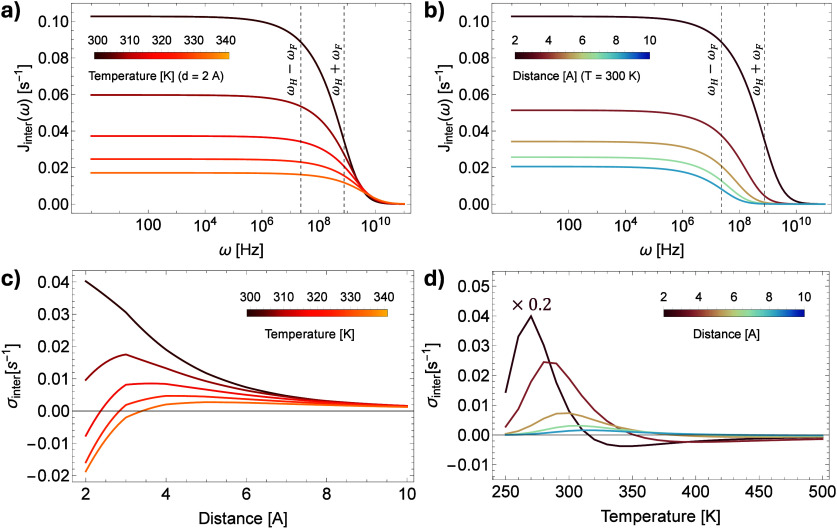
Spectral density frequency profile describing the intermolecular heteronuclear ^1^H–^19^F dipolar interaction at different temperatures (a) and intrapair distances (b). The [BMIM][PF6] self-diffusion rates were chosen for calculating the translational correlation time from eq 2. Inset: Sum and differences of ^1^H–^19^F Larmor frequencies at 9.4 T. Distance (c) and temperature (d) profiles of the intermolecular cross-relaxation rate highlighting how closer shells significancy change their contribution with increasing temperature compared to more distant shells.

As spins are not distributed uniformly, we scale the distance-dependent contributions with the RDF profile *g*_*HF*_(*r*) (Figure 5a) in order to account for the actual spatial distributions of nuclei within ionic liquids,^16,30,31^ in this case [BMIM][PF_6_]. When examining a cumulative integral, we obtain the ensemble cross-relaxation rate to which all spins within *r*_max_ distance contribute as . Figure 5b shows the convergence of these integrals at different temperatures, highlighting that above 330 K, closer shells contribute with negative values to σ, while distant shells increase the overall cross-relaxation rate due to their positive contributions. For comparison, we calculated the cross-relaxation rates σ_*H*2-*F*_^*MD*^(*r*_*max*_) from MD simulations by considering the neighboring ^19^F spins surrounding H2 protons up to the first, second, third, fourth and fifth local minima of the RDF plot (Figure 5c). These minima represent the spatial boundaries of the corresponding shells. A similar trend is observed: by considering more distant spins, the cross-relaxation rates converge to higher (more positive) values. Also, above 330 K, the negative σ values given by the closer shells decrease and become positive when considering more distant spins.

**Figure 5:**
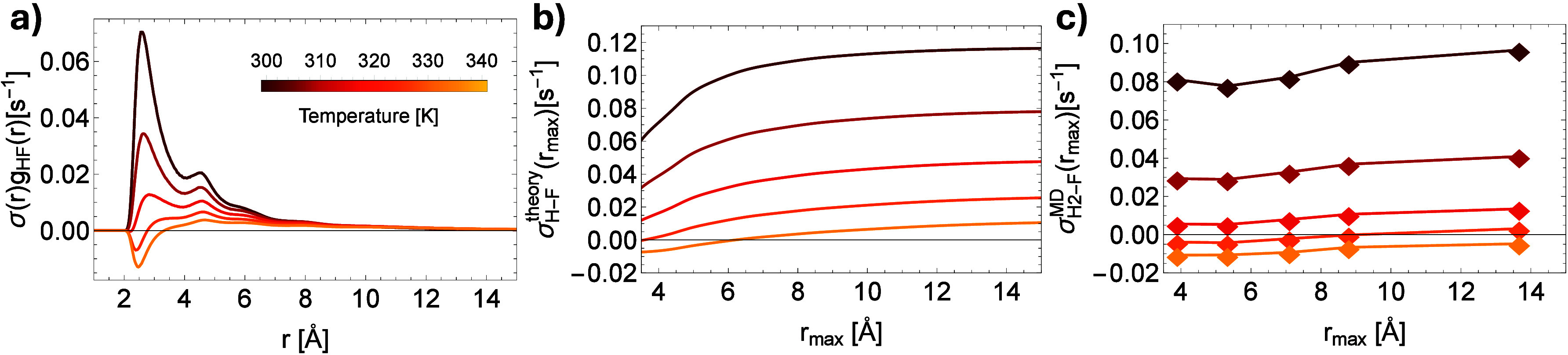
(a) Distance dependence of RDF-scaled cross-relaxation rate contributions at different temperatures. The closest shells contribute the most to the overall cross-relaxation rate and their contribution is significantly altered by temperature. The distant shells contribute to a smaller degree and are insensitive to temperature. (b) Convergence of the cumulative integral σ_*H*-*F*_^*theory*^(*r*_*max*_) as a function of the maximum distance up to which the shells’ contributions are considered. (c) Convergence of MD-calculated cross-relaxation rates σ_*H*2-*F*_^*MD*^(*r*_*max*_) at different temperatures with increasing distance up to which spins are considered. Distances correspond to the position of local minima in the RDF plot (Figure 3a).

These findings highlight the different shell-resolved contributions to the cross-relaxation rate as well as their intricate temperature dependency. As a corollary, the observed cross-relaxation rate is a sum of successive shell contributions: at the lowest temperatures, closer spins account for most of the cross-relaxation rate. Their contributions are significantly impacted by the temperature, while distant shells contribute to a smaller degree and are less sensitive to changes in temperature. One further important observation is that when a null intermolecular cross-relaxation rate is measured, it is the result of the mutual cancellation of shell contributions (within the first and second coordination shells), underscoring that different coordination shells operate under different motional regimes.

In conclusion, the temperature dependence of intermolecular cross-relaxation rates is influenced primarily by dynamical aspects, and structural changes with temperature do not appear to play a role. The relative order of σ among BMIM’s ^1^H spin system is due to the different proximity of fluorine atoms, while changes due to increasing temperature are caused by higher translational diffusion rates which modulate the translational correlation time. The observed cross-relaxation rate is a sum of successive shell contributions, where the first two shells contribute the most and are significantly impacted by temperature, while the distant nuclei only contribute to a smaller extent. Different shells are further characterized by different motional regimes to the extent that the cross-relaxation rates may change sign.

Understanding the different intermolecular factors that govern IL dynamics is important for the rational design of such systems. The fact that structural factors are of little importance for the temperature behavior and dynamical contributions (notably diffusion coefficients) are most important for the intermolecular interactions provides a simple guide toward tailoring systems for specific needs. For example, one may consider specifically tailoring systems that break out of such a regime by crossing a potential barrier at a specific temperature to achieve the desired properties. Another important aspect that can be explored by HOESY and the identified models is the microheterogeneity^32^ of ionic liquids upon dissolution as dynamic domains containing ionic clusters can coexist with fully solvated ions. In such a case, a significant decrease of intermolecular Overhauser transfer is expected, while in clusters the transfer can provide information about their size and tumbling rates. Previous MD studies^33,34^ showed that ion pairs are prevalent in low concentration ILs dissolved in solvents with low dielectic constants, while in neat samples, the time scale of the anion–cation interactions ranges from several picoseconds (hydrogen bond cleavage) to a few nanoseconds (Coulombic interactions) with no significant neutral ionic clusters diffusing together. These dynamical regimes can be studied using NMR by analyzing either the polarization lifetime or the intermolecular Overhauser transfer as described in this work.

## Methods

### Sample Preparation

The ionic liquids, 1-butyl-3-methylimidazolium tetrafluoroborate ([BMIM][BF_4_]) and 1-butyl-3-methylimidazolium hexafluorophosphate ([BMIM][PF_6_]), were prepared for NOE measurement in this experiment. Each ionic liquid was transferred into the inner tube of a 5 mm coaxial NMR tube, while deuterated dimethyl sulfoxide (DMSO) was added to the outer layer of each coaxial tube for the field lock. The sample preparation was performed inside an argon-filled glovebox to maintain an anhydrous and oxygen-free environment, preventing the hydrolytic degradation of the ionic liquids and excluding any possibility of water contamination that could interfere with the NMR results.

### Longitudinal Relaxation Rate (*R*_1_) Measurements

The longitudinal relaxation time (*T*_1_) of ^1^H and ^19^F in the ILs were measured on Bruker Avance III 400 NMR spectrometer. The experimental temperature ranged from 300 to 340 K with increments of 10K. The inversion recovery pulse sequence was used.

### ^1^H and ^19^F Cross Relaxation Rate (σ) Measurements

The ^1^H and ^19^F cross relaxation rates (σ) were determined in the HOESY experiments at the same temperatures with the *R*_1_ measurements. The experiments were performed at a ^1^H Lamor frequency of 400 MHz on a Bruker Avance III 400 NMR spectrometer (9.4T). In this experiment the nonselective version of the 1-D HOESY pulse sequence was used as described by Combettes et al.^23^ with ^1^H detection. The 1D HOESY experiment is time-efficient and effective especially since in the current application we only have one ^19^F resonance. The sequence begins with two 90° fluorine pulses to either invert the spins or flip them back in alternate steps of the phase cycle (the phase cycle is shown in Figure 1). The sum between these two experiments will record the magnetization transfer due to ^19^F–^1^H cross-relaxation during the subsequent mixing time τ. The initial proton magnetization is purged using a 90° proton pulse combined with bipolar gradient pulses in the Z direction with 15% strength (approximately 7.5 G/cm) and 1 ms duration. These gradients also remove any transverse magnetization from imperfect inversion pulses. At the midpoint of the mixing time, the 180° proton pulses are applied to maintain longitudinal proton magnetization close to zero, preventing proton relaxation from affecting NOE development. The sequence concludes with a final 90° proton read pulse to observe the developed NOEs. The final two 90° fluorine pulses serve to flip the magnetization back from the inverted state (to allow for a shorter recycle delay).

A single-pulse ^1^H NMR spectrum with 4 scans was also acquired to normalize HOESY signals. The receiver gain is set to 2 since the magnetization of protons is relatively large in neat IL samples. Since the NOE signal intensity builds up mostly linearly initially and subsequently decays exponentially, the integral of the proton signals in 1-D HOESY spectra was collected at mixing times from 1 to 5s with increments of 0.1s before 1s and 1s between 1s and 5s across various temperatures. The 90° pulses for ^1^H and ^19^F were 16.38 μs and 33 μs, respectively, while the recycle delay was 6 s.

### Molecular Dynamics Simulations

The molecular dynamics (MD) simulations were performed using GROMACS 2023.3 software package^35^ using a refined OPLS force field with scaled charges developed for IL.^36^ The simulation box was created using Packmol^37^ and consisted of 500 BMIM^+^ cations and 500 anions (either BF_4_^–^ or PF_6_^–^) with a volume chosen to match the densities of each IL. Periodic boundary conditions were applied to each box with long-range interactions (up to 12 Å) handled with Particle-Mesh Ewald summations. Initial energy minimization was performed by the steepest descent algorithm for 5000 steps. Equations of motion were integrated using the leapfrog algorithm with a time step of 1 fs. Equilibration was performed at different temperature values between 300 and 340 K and a constant pressure of 1.0 bar was maintained with the Berendsen coupling during an isothermal–isobaric ensemble (NPT) simulation for 5 ns of equilibration. The 40 ns production run were performed under NPT conditions storing coordinates every 25 ps. The MDAnalysis software package^38^ was used to compute radial distribution functions (RDFs), mean-square displacement (MSD) and extract coordinates from which the intermolecular ^1^H–^19^F dipolar interactions were evaluated. Cross-relaxation rates were estimated from MD trajectories using a custom PYTHON script. Spatial distribution functions (SDFs) were calculated using VIAMD software package^39^ to highlight BMIM’s interaction sites where anions are most likely to be attracted. SDFs represent a more informative three-dimensional extension of RDFs which accounts for directional correlation unlike the latter which assume an implicit spherical symmetry.^40^ All plots were created by using Mathematica.

## Supporting Information Available

Experimental autorelaxation rates (Figure S1), HOESY build-up points with fitted curves (Figure S2), and self-diffusivity computed from MD simulations (Figure S3) (PDF)
Mathematica notebook performing the analytical derivation of the cross-relaxation rate (ZIP)
Transparent Peer Review report available (PDF)
