# Supplementary material for: Molecular Shells and Range of Interactions in Ionic Liquids as a Function of Temperature
Source: J Phys Chem Lett. 2025 Feb 20;16(8):2120–7. doi: 10.1021/acs.jpclett.4c03576 (PMC11873955; doi:10.1021/acs.jpclett.4c03576)
Supplement: Supplementary file 1 — jz4c03576_si_001.pdf [file jz4c03576_si_001.pdf]

Supporting Information: Molecular shells and range of interactions in ionic liquids as a function of temperature

Zhiyuan Gao†, Florin Teleanu†, Kelsey Anne Marr, and Alexej Jerschow*

Department of Chemistry, New York University, New York, NY 10003, United States

E-mail: alexej.jerschow@nyu.edu

Experimental ^{1}H Auto-Relaxation Rates

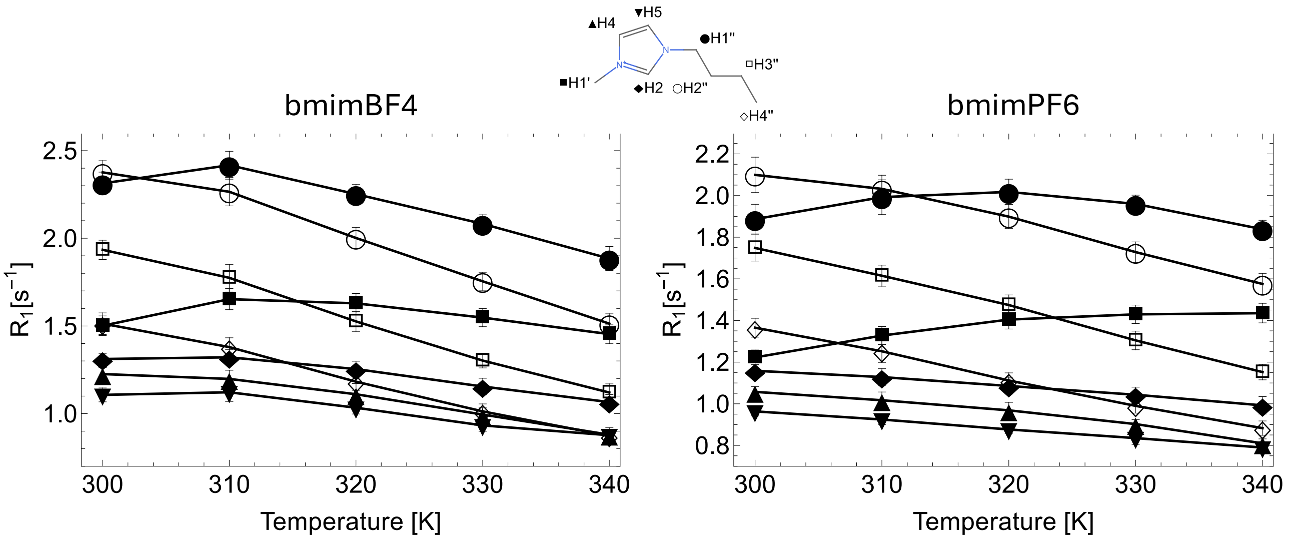

Figure S1: Auto-relaxation rates of ^{1}H spins in bmim cations for both ILs investigated. The rates were measured using inversion recovery sequence at different temperatures.

Fitting ^{1}H-detected 1D HOESY build-up curves

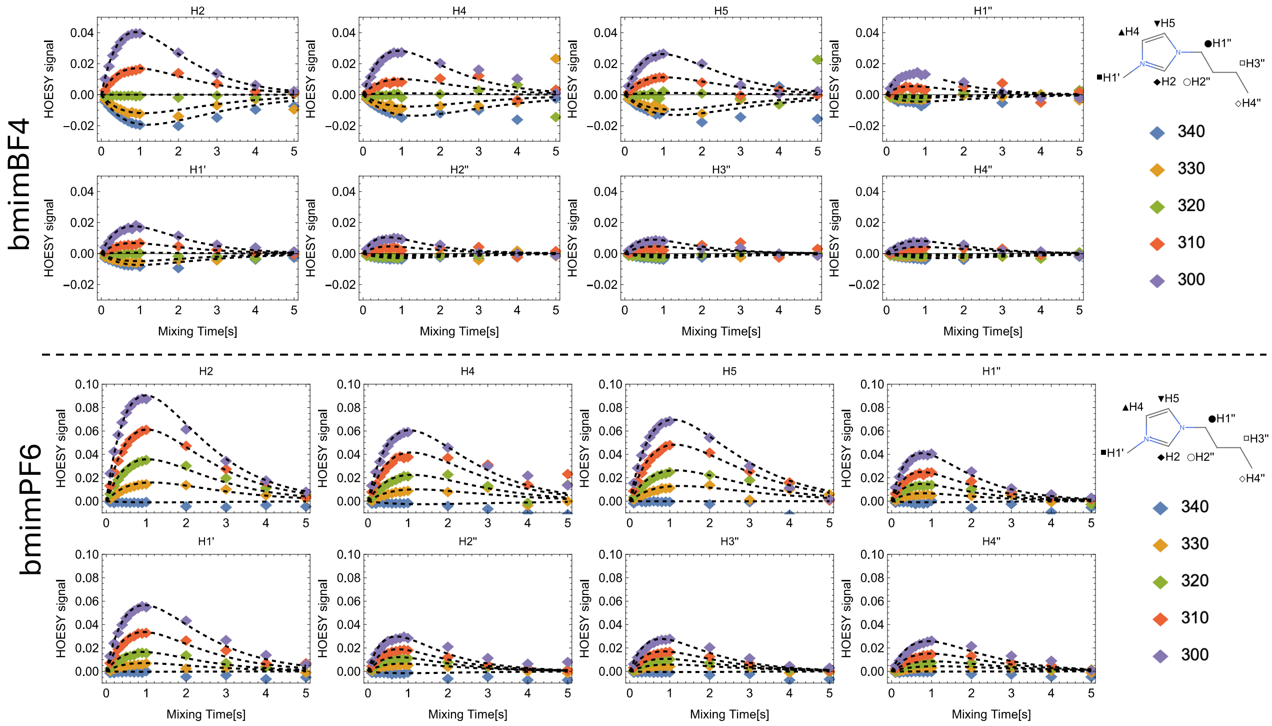

Figure S2: 1D HOESY build-up curves for selected ^{1}H spins in bmim for the two ILs at different temperatures. Using the pulse sequence from Figure 1a, the peak integrals at different mixing times are plotted as markers for all detected resonances in Figure 1b (proton labeling is shown on the right). The dashed lines are fitted functions using Equation 1 from the Main text. The cross-relaxation rates is the only fitting parameter and the values are plotted in Figure 1.

Comparison between experimental and MD-derived self-diffusivity of cations and anions in ILs

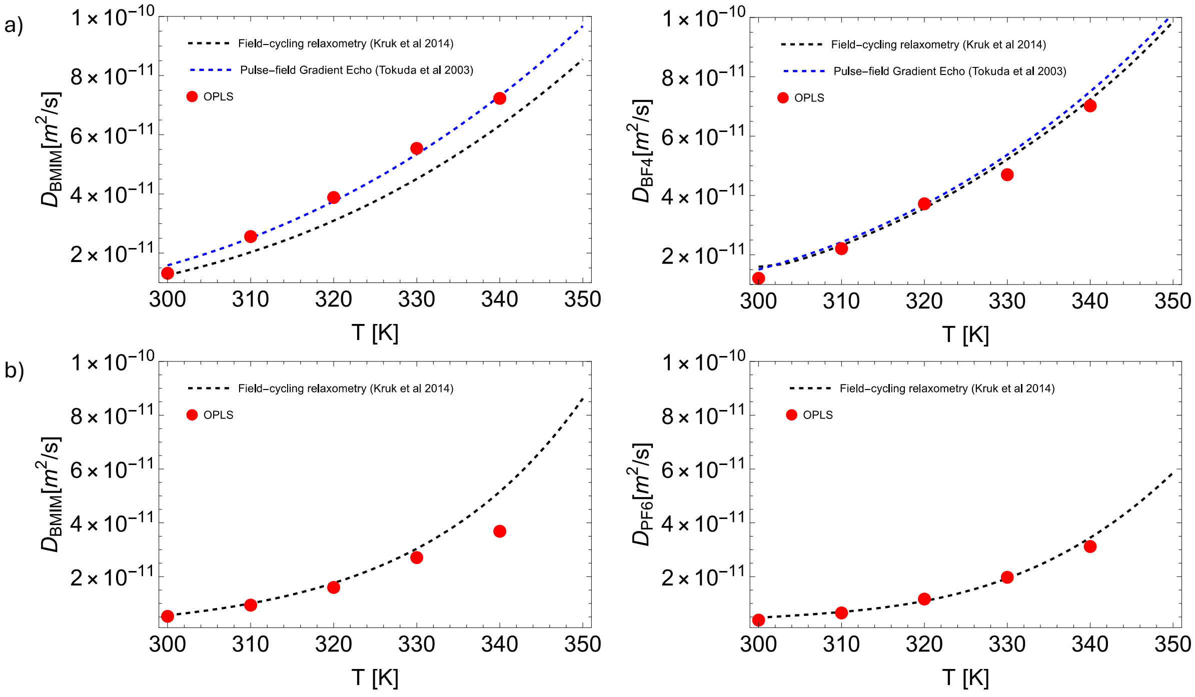

Figure S3: Self-diffusivity rates of cations and anions for bmimBF4 (a) and bmimPF6 (b) systems. Red points represent diffusion rates from molecular dynamics simulations (using OPLS with scaled charges[S1]) calculate by fitting the MSD plots from Figure 2b and 3b from the main text. Dashed lines are interpolated functions using the experimental diffusion rate values from either pulse-field-gradient spin-echo[S2] or field-cycling relaxometry[S3]

References

(S1) Doherty, B.; Zhong, X.; Gathiaka, S.; Li, B.; Acevedo, O. Revisiting OPLS Force Field Parameters for Ionic Liquid Simulations. *Journal of Chemical Theory and Computation* **2017**, *13*, 6131–6145.

(S2) Tokuda, H.; Hayamizu, K.; Ishii, K.; Susan, M. A. B. H.; Watanabe, M. Physico-

chemical Properties and Structures of Room Temperature Ionic Liquids. 1. Variation of Anionic Species. *The Journal of Physical Chemistry B* **2004**, *108*, 16593–16600.

(S3) Kruk, D.; Meier, R.; Rachocki, A.; Korpała, A.; Singh, R. K.; Rössler, E. Determining diffusion coefficients of ionic liquids by means of field cycling nuclear magnetic resonance relaxometry. *Journal of Chemical Physics* **2014**, *140*, 244509.
