# Supplementary material for: Molecular Shells and Range of Interactions in Ionic Liquids as a Function of Temperature
Source: J Phys Chem Lett. 2025 Feb 20;16(8):2120–7. doi: 10.1021/acs.jpclett.4c03576 (PMC11873955; doi:10.1021/acs.jpclett.4c03576)
Supplement: Supplementary file 3 — jz4c03576_si_003.pdf [file jz4c03576_si_003.pdf]

jz-2024-03576k.R1

Name: Peer Review Information for "Molecular shells and range of interactions in ionic liquids as a function of temperature"

First Round of Reviewer Comments

Reviewer: 1

Comments to the Author

The dynamics of two ionic liquids, [BMIM][BF4] and [BMIM][PF6], were investigated by NMR spectroscopy in this work. The authors found that the intermolecular H-F spin coupling constants change their signs within a limited range of temperature, and ascribed the temperature dependence to the translational dynamics of ions. The authors also performed MD simulation of the same systems, and succeeded in reproducing the characteristic temperature dependence of the spin coupling constant.

The NMR spectroscopy seemed to have been performed properly, and the results were reproduced well by MD simulation. The interpretation of the results was based on both experiment and simulation, and it sounds physically reasonable. However, the physical insight obtained in this work is on the NMR spectroscopy, not on the ionic liquids. The observed characteristic behavior of the coupling constant is because the time scale of the dynamics of the two ionic liquids happened to match that of the H-F spin coupling of the spectrometer used in this experiment. This work thus attracts only the limited range of physical chemists, and I recommend the editor to transfer this manuscript to the Journal of Physical Chemistry B, rather than publishing it in the Journal of Physical Chemistry Letters. Comments on detailed points are itemized below.

1. In the last sentence of the second paragraph, the authors ascribed the larger chemical shifts of the protons near the imidazolium ring to the higher positive charges on these protons. I admit that the lower electron density is an important reason, but the effect of the ring current should not be neglected.

2. In the second paragraph of page 6, I recommend the authors to show the values of omega_H and omega_F in order to provide the information on the time scale of the spin coupling.

3. In the first line of page 7, the approximation of D_HF = D_H + D_F assumes the independent diffusional motions of the two atoms, which is doubtful for the adjacent pair of ions. I guess that the breakdown of this assumption may explain the difference between Figs. 4b and 4c.

4. In eqs. 3 and 4, the authors consider the spin coupling between individual H and F atoms. However, the spins of the equivalent sites (for example, four F-atoms of BF4 and six F-atoms of PF6)

are actually coupled. How do the authors consider the influence of this intramolecular spin coupling?

5. In the second paragraph of page 10, the authors described that the distance-dependent coupling constant was evaluated using eq. 2. However, I cannot find r (spin-spin distance) in eq. 2.

6. Page 9, the fifth-last line: “cross-relation rates”

Reviewer: 2

Comments to the Author

Ga et al. describe the use of temperature-dependent HOESY to extract the cross-relaxation rate between fluorine (F) and hydrogen (H) cations. The solvation structure of two ionic liquids (ILs) is discussed, and molecular dynamics (MD) simulations are conducted to further support and provide more details on the interactions between H and F in different ILs. Overall, this is an interesting study utilizing NMR to investigate anion-cation interactions. I recommend it for publication after addressing the following concerns.

Major concerns:

1. The cross-relaxation rate changes sign with increasing temperature. It is important to provide a rationale for this behavior. On Page 7, Line 18, the explanation of how the correlation time module affects the sign of zero and double quantum contributions is unclear mathematically. A more detailed explanation would be helpful.

2. RDF can be obtained from MD simulations. Is it possible to measure the H-F distance in this IL system experimentally?

3. Although the importance of understanding anion-cation interactions is mentioned in the introduction, the conclusion does not provide a detailed discussion on how the findings of this paper contribute to the advancement of future applications of ionic liquids. While this is not the main focus of the work, the reviewer suggests that further discussion on this topic could attract broader attention from readers.

Minor points:

1. The error bars in Figure 1 should be clarified.

2. Although the proximity map is mentioned, it would be helpful to briefly explain how the map is constructed and what it reveals about molecular interactions.

Author's Response to Peer Review Comments:

Reviewer 1 Comments:

The dynamics of two ionic liquids, [BMIM][BF4] and [BMIM][PF6], were investigated by NMR spectroscopy in this work. The authors found that the intermolecular H-F spin coupling constants change their signs within a limited range of temperature, and ascribed the temperature dependence to the translational dynamics of ions. The authors also performed MD simulation of the same systems, and succeeded in reproducing the characteristic temperature dependence of the spin coupling constant.

The NMR spectroscopy seemed to have been performed properly, and the results were reproduced well by MD simulation. The interpretation of the results was based on both experiment and simulation, and it sounds physically reasonable. However, the physical insight obtained in this work is on the NMR spectroscopy, not on the ionic liquids. The observed characteristic behavior of the coupling constant is because the time scale of the dynamics of the two ionic liquids happened to match that of the H-F spin coupling of the spectrometer used in this experiment. This work thus attracts only the limited range of physical chemists, and I recommend the editor to transfer this manuscript to the Journal of Physical Chemistry B, rather than publishing it in the Journal of Physical Chemistry Letters. Comments on detailed points are itemized below.

We appreciate reviewer's insights and suggestions regarding our work which we believe can attract the interest of researchers beyond the NMR field as the intermolecular interactions studied herein are a powerful, yet simple tool to study ion pairing or clustering in ionic liquids which are known to exhibit dynamic microheterogeneity (Wang et al. 2020 Chem. Rev.) impacting their transport properties such as viscosity and electrical conductivity. The ability to transfer polarization between ions should be significantly hampered in a well dispersed system such as ionic liquids dissolved in other solvents. However, if ions are not fully solvated, but aggregate in discrete nanometer size clusters, Overhauser transfer is still possible in such conditions and can point to the presence of heterogeneous domains. Previous MD studies (Zhao et al JACS 2009) showed that the ionic contacts persist for several nanoseconds which is on proper timescale for NMR relaxometry studies. Understanding temperature and distance behavior of these interactions can aid the interpretation of experimental data and further derivation of ionic cluster size and tumbling rates. NMR spectroscopy and the techniques used in this work are the only tools for the dynamic characterization of the system.

1. In the last sentence of the second paragraph, the authors ascribed the larger chemical shifts of the protons near the imidazolium ring to the higher positive charges on these protons. I admit that the lower electron density is an important reason, but the effect of the ring current should not be neglected.

Indeed, the ring current effects in the imidazolium unit plays a significant role in shifting proton's chemical shifts to lower frequencies and accounts for the observed spectral differences between the aliphatic protons and the ones experiencing the deshielding field

induced by electronic currents. Our intention was to highlight how chemical shift, electric charges and ion contacts are correlated as more positively-charged sites will attract more anions and will also lead to enhanced deshielding effects. We added another sentence addressing this aspect.

2. In the second paragraph of page 6, I recommend the authors to show the values of omega_H and omega_F in order to provide the information on the time scale of the spin coupling.

Thank you for this suggestion. We added the values of the Larmor frequencies for both 1H and 19F spins.

3. In the first line of page 7, the approximation of D_HF = D_H + D_F assumes the independent diffusional motions of the two atoms, which is doubtful for the adjacent pair of ions. I guess that the breakdown of this assumption may explain the difference between Figs. 4b and 4c.

Indeed, diffusion in ionic liquids is a complex phenomenon due to long-range electrostatic interactions and the similar diffusion regimes of the two ions in both [BMIM][BF4] and [BMIM][PF6]. The assumption we use in Equation 2 and Figure 4b implies that the relative diffusion (D_{HF}) is independent of inter-ionic distances which might not be the case for the MD results shown in Figure 4c. Ionic liquids are known to exhibit dynamics ranging from ballistic motion at short times, to "cage escape" dynamics at longer times and finally to linear time dependence of steady-state diffusion regime. Previous MD studies (Zhao et al JACS 2009) showed that close ions can interact via hydrogen bonds or short-range electrostatic interactions on different timescales. Time correlation functions characterizing hydrogen bond decorrelates within few picoseconds, while ion contacts persist for nanoseconds. However, translational diffusion of stable ionic pairs was shown to be insignificant in several studies (Zhao et al JACS 2009, Kirchner et al J. Phys.: Condens. Matter 2015). We have relevant references and a statement about this to the revised version.

4. In eqs. 3 and 4, the authors consider the spin coupling between individual H and F atoms. However, the spins of the equivalent sites (for example, four F-atoms of BF4 and six F-atoms of PF6) are actually coupled. How do the authors consider the influence of this intramolecular spin coupling?

Indeed, the chemically and magnetically equivalent fluorine atoms of the same ion interact via the bond-mediated scalar coupling or J-coupling. This interaction depends only on the wave-function describing the anion and not on the molecular tumbling as in the case of intermolecular cross-relaxation rate presented in our work. As the intermolecular transfer

involves the sum of fluorine longitudinal polarization $\sum_i F_z^i$ to BMIM proton longitudinal polarization H_z^j and due to the commutation relation $\left[\sum_i F_z^i, \sum_{i,j} J_{FF} \vec{F}^i \cdot \vec{F}^j\right] = 0$, the fluorine-fluorine J coupling does not contribute to the spin dynamics in our experiments.

5. In the second paragraph of page 10, the authors described that the distance-dependent coupling constant was evaluated using eq. 2. However, I cannot find r (spin-spin distance) in eq. 2.

The distance dependency of spectral density in Equation 2 is given by the distance of closest approach d that resides both inside and outside the integral, scaling the intensity of the spectral density as well as the translational correlation time $\tau_c = d^2/(D_H + D_F)$

6. Page 9, the fifth-last line: “cross-relation rates”

We corrected this error.

Reviewer 2’ comments

Ga et al. describe the use of temperature-dependent HOESY to extract the cross-relaxation rate between fluorine (F) and hydrogen (H) cations. The solvation structure of two ionic liquids (ILs) is discussed, and molecular dynamics (MD) simulations are conducted to further support and provide more details on the interactions between H and F in different ILs. Overall, this is an interesting study utilizing NMR to investigate anion-cation interactions. I recommend it for publication after addressing the following concerns.

Major concerns:

1. The cross-relaxation rate changes sign with increasing temperature. It is important to provide a rationale for this behavior. On Page 7, Line 18, the explanation of how the correlation time module affects the sign of zero and double quantum contributions is unclear mathematically. A more detailed explanation would be helpful.

We added further explanations on how temperature and distance of closest approach impacts spectral densities characterizing the intermolecular dipolar coupling and what is the expected trend of cross-relaxation rate on a much larger temperature range.

2. RDF can be obtained from MD simulations. Is it possible to measure the H-F distance in this IL system experimentally?

The H-F distances are dynamically changing due to molecular collisions and the Radial Distribution Functions provides average probability distributions of these distances. The structure factor S(q) could be measured by x-ray/neutron scattering experiments in liquids, and the g(r) function can be derived by Fourier Transformation. The limited structural information of intermolecular HOESY experiments, compared to the intramolecular case where fixed distances can be measured, is a known issue and our work adds new insight into how different shells contribute to the observed cross-relaxation rate. We note that our work focused on the dynamical aspects of the interactions, which would not be easily assessed by neutron diffraction.

3. Although the importance of understanding anion-cation interactions is mentioned in the introduction, the conclusion does not provide a detailed discussion on how the findings of this paper contribute to the advancement of future applications of ionic liquids. While this is not the main focus of the work, the reviewer suggests that further discussion on this topic could attract broader attention from readers.

We believe our work can attract the interest of researchers beyond the NMR field as the intermolecular interactions studied herein are a powerful, yet simple tool to study ion pairing or clustering in ionic liquids which are known to exhibit dynamic microheterogeneity (Wang et al. 2020 Chem. Rev.) impacting their transport properties such as viscosity and electrical conductivity. The ability to transfer polarization between ions should be significantly hampered in a well dispersed system such as ionic liquids dissolved in other solvents. However, if ions are not fully solvated, but aggregate in discrete nanometer size clusters, Overhauser transfer is still possible in such conditions and can point to the presence of heterogeneous domains. Understanding temperature and distance behavior of these interaction can aid the interpretation of experimental data and will help establishing guidelines for the development of ionic liquids with specific desired qualities. We have added a statement to the conclusions accordingly.

Minor points:

1. The error bars in Figure 1 should be clarified.

We added further clarifications on how the error bars were calculated.

2. Although the proximity map is mentioned, it would be helpful to briefly explain how the map is constructed and what it reveals about molecular interactions.

We added a brief description of Spatial Distribution Function (SDF) in the 'Methods' section. SDFs represents a 3D extension of Radial Distribution Functions (RDFs) showing

here the isosurfaces of the probability density for finding the anion around the different sites of the cation. SDF accounts for directional correlation unlike the monodimensional RDF which assume an implicit spherical symmetry. Thus, SDF is more informative regarding interaction sites. In our paper, the isosurface SDF plot shows the specific protons around which anion will likely reside around the positively charged imidazolium ring which is in perfect agreement with the measured cross-relaxation rates.

jz-2024-03576k.R2

Name: Peer Review Information for "Molecular shells and range of interactions in ionic liquids as a function of temperature"

Second Round of Reviewer Comments

Reviewer: 1

Comments to the Author

In the reply to my comment on the broad interest, the authors wrote that the NMR spectroscopy is the only tool for the dynamic characterization of the present system in the hundreds MHz regime. However, quasi-elastic neutron scattering spectroscopy has also been applied to various ionic liquids to resolve the dynamics in the sub-ns regime. I appreciate this work as an excellent application of NMR technique, and I would like to ask the editor to judge whether it has the broad interest for the publication in the Journal of Physical Chemistry Letters.

The replies and the revisions to my comments on detailed points are satisfactory except for those on my comment 4. In the presence of intramolecular J-coupling of F-atoms in an anion, an H-atom interact with the sum of the F-spins within an anion, rather than with an individual F-spin. Therefore, in the calculation of the time correlation function G(t) in eq. (3), the cross correlation between different H-F pairs should also be taken into account. Whether the cross correlation is present or not affects the contribution of the reorientational motion of an anion to G(t). I recommend the authors to add a comment on this point prior to publication.

Author's Response to Peer Review Comments:

Reviewer 1 Comments:

In the reply to my comment on the broad interest, the authors wrote that the NMR spectroscopy is the only tool for the dynamic characterization of the present system in the hundreds MHz regime. However, quasi-elastic neutron scattering spectroscopy has also been applied to various ionic liquids to resolve the dynamics in the sub-ns regime.

Response: We thank the reviewer for pointing this out, and we changed the wording accordingly. We wish to point out that while QENS is very powerful, it is significantly less available as a technique. We added a short note about this as well.

[… regarding previous reply to comment 4:] In the presence of intramolecular J-coupling of F-atoms in an anion, an H-atom interact with the sum of the F-spins within an anion, rather than with an individual F-spin. Therefore, in the calculation of the time correlation function G(t) in eq. (3), the cross correlation between different H-F pairs should also be taken into account. Whether the cross correlation is present or not affects the contribution of the reorientational motion of an anion to G(t). I recommend the authors to add a comment on this point prior to publication.

Response: We thank the reviewer for this excellent observation, and although such effects were not detectable in our data (the spin-pairs could be analyzed independently), we acknowledge the possibility of the contribution of cross-correlated DD-DD interactions to the observed cross-relaxation rates. In response to the reviewer's comment, we derived the full analytical expression of the cross-relaxation rate from the sum of 19F longitudinal magnetization to the longitudinal magnetization of a neighboring 1H spin. We achieved this using the automated symbolic processing of Bloch-Redfield-Wangsness relaxation theory workflow described in Kuprov et al JMR 2007 for a system containing one 1H and four 19F where all dipolar couplings and chemical shield anisotropy (CSA) interactions and all their cross-correlations are considered. The result proves that the cross-relaxation rate from $\sum_{"} F_{!}^{"}$ to $H_{!}$ depends only on the pair-wise 1H-19F interactions and no cross-correlated mechanism is involved. However, cross-correlated interactions are responsible for more complex polarization transfers such as the one $F_{!}^{\#}F_{!}^{\$}H_{!}$ to $H_{!}$ as shown in the attached notebook as Supporting Information, which, however, is not detectable with the current methods. Also, we added a small paragraph to the main text regarding these results. Thank you again for this suggestion!
